# Multiple familial trichoepithelioma: report of a disfiguring case^⋆^

**DOI:** 10.1016/j.abd.2022.11.007

**Published:** 2024-02-24

**Authors:** Thais Florence Duarte Nogueira, Stefanie Gallotti Borges Carneiro, Larissa Jacom Abdulmassih Wood, José Roberto Pereira Pegas

**Affiliations:** Service of Dermatology, Complexo Hospitalar Padre Bento de Guarulhos, Guarulhos, SP, Brazil

Dear Editor,

This case describes a 40-year-old woman, with normochromic asymptomatic papules and nodules on the face, scalp, and upper back since puberty, which gradually increased in number and size (Fig. 1).

**Figure 1:**
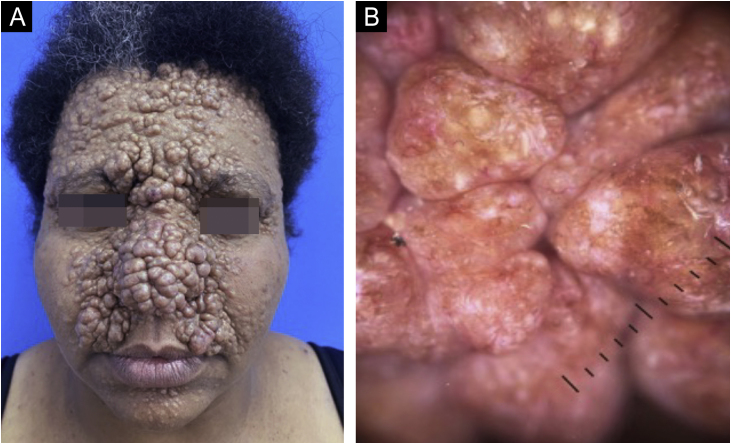
(A) Multiple confluent, normochromic, shiny, well-defined papules and nodules, measuring 0.5 to 2 cm in diameter, located predominantly and symmetrically on the central face; (B) Dermoscopy: arboriform vessels, chrysalises and milium pseudocysts over pink areas.

She reported difficulty in getting a job due to the skin lesions. She denied other comorbidities and medication use and reported that other family members had similar, but less extensive lesions (Fig. 2).

**Figure 2:**
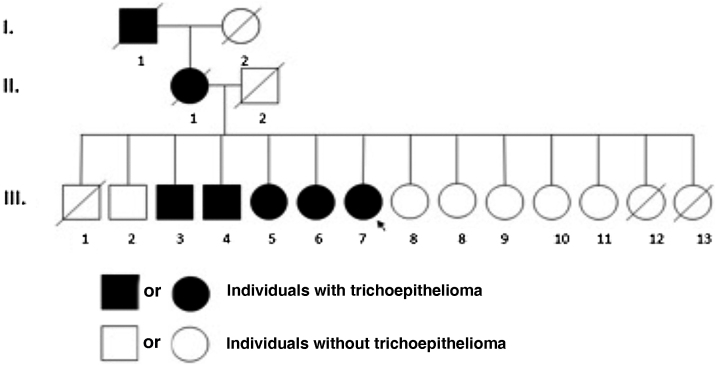
Pedigree showing autosomal dominant inheritance pattern of multiple familial trichoepithelioma.

Dermoscopy (Fig. 1) and histopathology (Fig. 3) were compatible with trichoepitheliomas. It was not possible to perform genetic testing due to its unavailability.

**Figure 3:**
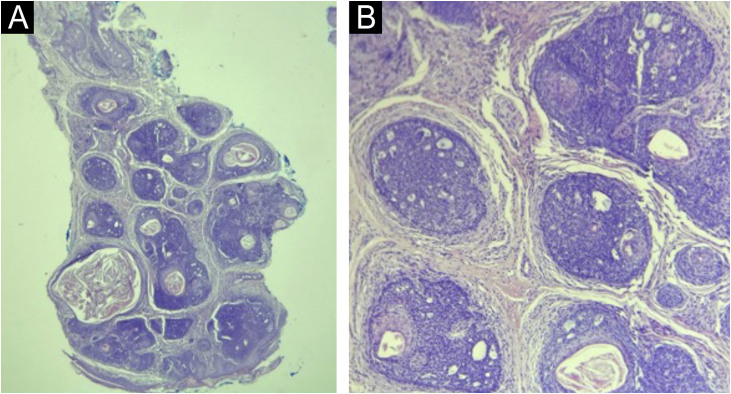
(A) Histopathology: well-defined dermal tumor (trichoepithelioma), consisting of lobules of basaloid cells and focal keratin pseudocysts (Hematoxylin & eosin, ×100). (B) Lobules of basaloid cells, with a peripheral palisade and cribriform pattern, surrounded by fibrotic stroma, associated with focal Keratin pseudocysts. There is direct contact between stroma and tumor cells (Hematoxylin & eosin, ×200).

Treatment was administered with imiquimod 5% cream twice a day and 0.5% topical tretinoin at night for six months, progressing with skin irritation and without significant improvement of the lesions.

Multiple familial trichoepithelioma (MFT) is a rare autosomal dominant genodermatosis associated with genetic mutations in the tumor suppression and cylindromatosis (CYLD) genes located, respectively, on chromosomes 9p21 and 16q12-q13.1-3. These genes favor the proliferation and differentiation of the germ cells of the pilosebaceous units, enabling the development of trichoepitheliomas.^1^, ^2^

MFT affects more women due to its lower expressiveness and genetic penetrance in males.^1^, ^2^ There is no racial predilection^3^ and a family history is generally positive.^1^, ^2^ It manifests in childhood or adolescence, with the appearance of papulonodular, normochromic, or erythematous, shiny lesions, which mainly affect symmetrically the central region of the face.^1^, ^2^ They can also affect the scalp, cervical region, and upper chest.^1^, ^2^ Over the years, the lesions can increase in number and size.^1^, ^2^

The phenotype in MFT is variable.^4^ While there are patients with few lesions, others have multiple confluent and deforming lesions.^4^ This generates important aesthetic consequences and psychosocial suffering.^1^, ^3^ Malignant transformation of trichoepitheliomas into trichoblastic or basal cell carcinoma may also occur, although it is rare.^1^, ^2^

The dermoscopy of trichoepitheliomas shows small, fine-caliber arboriform vessels, chrysalises and milium pseudocysts over white, pink and, less frequently, yellowish or brown areas.^3^

Histopathology indicates the presence of keratin pseudocysts and lobules of monomorphic basaloid cells arranged in a cribriform pattern, surrounded by abundant fibrous stroma.^1^, ^2^

The diagnosis of MFT depends on clinical-histopathological findings.^3^, ^5^ The genetic study is useful for counseling, but not essential.^3^, ^5^

Several syndromes present with facial papules and nodules, such as Brooke-Spiegler, familial cylindromatosis, Bazex-Dupré-Christol and tuberous sclerosis.^3^, ^5^ Hence, both clinically and histopathologically, it is important to rule out other associated complications that indicate another diagnosis.

Both MFT and familial cylindromatosis (FC) are considered different spectrums of Brooke-Spiegler syndrome (BSS), due to the mutation in the common CYLD.^4^ However, in BSS there are multiple spiradenomas, cylindromas, and trichoepitheliomas, while in FC there are only cylindromas, and in MFT, only trichoepitheliomas.^4^

The treatment of MFT is not well established in the literature and is difficult due to the multiplicity of lesions, the predominantly facial location, and the progressive nature of the lesions. Among available treatments, surgical excision, radiofrequency ablation, dermabrasion, cryotherapy, radiotherapy, and lasers stand out.^5^ There have also been reports of pharmacological therapies with sirolimus, imiquimod, tretinoin, vismodegib, acetylsalicylic acid and adalimumab.^5^

## Financial support

None declared.

## Authors' contributions

Thais Florence Duarte Nogueira: Design and planning of the study; collection of data; drafting and editing of the manuscript and critical review of important intellectual content; intellectual participation in the propaedeutic and/or therapeutic conduct of the studied cases; critical review of the literature; approval of the final version of the manuscript.

Stefanie Gallotti Borges Carneiro: Collection of data; drafting and editing of the manuscript and critical review of important intellectual content; approval of the final version of the manuscript.

Larissa Jacom Abdulmassih Wood: Collection of data; drafting and editing of the manuscript and critical review of important intellectual content; approval of the final version of the manuscript.

José Roberto Pereira Pegas: Design and planning of the study; collection of data; drafting and editing of the manuscript; critical review of important intellectual content; effective participation in research orientation; intellectual participation in the propaedeutic and/or therapeutic conduct of the studied cases; critical review of the literature; approval of the final version of the manuscript.

## Conflicts of interest

None declared.
